# Arabidopsis latent virus 1, a comovirus widely spread in *Arabidopsis thaliana* collections

**DOI:** 10.1111/nph.18466

**Published:** 2022-09-22

**Authors:** Ava Verhoeven, Karen J. Kloth, Anne Kupczok, Geert H. Oymans, Janna Damen, Karin Rijnsburger, Zhang Jiang, Cas Deelen, Rashmi Sasidharan, Martijn van Zanten, René A. A. van der Vlugt

**Affiliations:** ^1^ Laboratory of Virology Wageningen University and Research Droevendaalsesteeg 1 6708PB Wageningen the Netherlands; ^2^ Plant‐Environment Signaling Utrecht University Padualaan 8 3584 CH Utrecht the Netherlands; ^3^ Plant Stress Resilience Utrecht University Padualaan 8 3584 CH Utrecht the Netherlands; ^4^ Laboratory of Entomology Wageningen University and Research Droevendaalsesteeg 1 6708PB Wageningen the Netherlands; ^5^ Bioinformatics Group Wageningen University and Research Droevendaalsesteeg 1 6708PB Wageningen the Netherlands; ^6^ Molecular Plant Physiology Utrecht University Padualaan 8 3584 CH Utrecht the Netherlands; ^7^ Biointeractions and Plant Health Wageningen Plant Research Droevendaalsesteeg 1 6708PB Wageningen the Netherlands

**Keywords:** Arabidopsis latent virus 1 (ArLV1), *Arabidopsis thaliana*, comovirus, drought resilience, RNA sequencing, sequence read archives

## Abstract

- Transcriptome studies of Illumina RNA‐Seq datasets of different *Arabidopsis thaliana* natural accessions and T‐DNA mutants revealed the presence of two virus‐like RNA sequences which showed the typical two‐segmented genome characteristics of a comovirus.
- This comovirus did not induce any visible symptoms in infected *A. thaliana* plants cultivated under standard laboratory conditions. Hence it was named Arabidopsis latent virus 1 (ArLV1). Virus infectivity in *A. thaliana* plants was confirmed by quantitative reverse transcription polymerase chain reaction, transmission electron microscopy and mechanical inoculation. Arabidopsis latent virus 1 can also mechanically infect *Nicotiana benthamiana*, causing distinct mosaic symptoms.
- A bioinformatics investigation of *A. thaliana* RNA‐Seq repositories, including nearly 6500 Sequence Read Archives (SRAs) in the NCBI SRA database, revealed the presence of ArLV1 in 25% of all archived natural *A. thaliana* accessions and in 8.5% of all analyzed SRAs. Arabidopsis latent virus 1 could also be detected in *A. thaliana* plants collected from the wild.
- Arabidopsis latent virus 1 is highly seed‐transmissible with up to 40% incidence on the progeny derived from infected *A. thaliana* plants. This has probably led to a worldwide distribution in the model plant *A. thaliana* with as yet unknown effects on plant performance in a substantial number of studies.

## Abstract 2

See also the Commentary on this article by Sáez & Pagán, **237**: 1071–1073.

## Introduction

Many plant biology studies involve *Arabidopsis thaliana* as model plant. Although community‐serving *A. thaliana* stock centers, such as the Arabidopsis Biological Resource Centre (ABRC) (Scholl & Anderson, 1994), test for seed‐borne diseases, usually only visual detection methods and germination tests are applied (Rivero *et al*., 2014). Moreover, few research laboratories have reported testing their seed stocks for known virus contaminations before running experiments. The *A. thaliana* genome sequence became publicly available in 2000 (The Arabidopsis Genome Initiative, 2000). Since then, whole‐transcriptome sequencing has become one of the most common tools for deciphering plant physiological processes. This untargeted approach can also reveal the unexpected presence of other biological agents and provides information about possible unnoted infections and contaminations (Villamor *et al*., 2019).

Viruses, in particular, can be hiding in plants and in seed material (Cobos *et al*., 2019). The majority of well‐studied viruses cause disease symptoms in agriculturally important crops with sometimes severe effects on plant morphology, physiology and yield (Prasad *et al*., 2020). In nature, however, plants are often infected with viruses that do not cause any apparent disease symptoms, so‐called latent infections (Shates *et al*., 2019). Many viruses may even be beneficial to their hosts in a mutualistic symbiosis (Roossinck, 2011). Extending the knowledge on these latent viruses will contribute to the beneficial exploitation of viruses in cultivated crops (Takahashi *et al*., 2019).

Here, we describe a comovirus; Arabidopsis latent virus 1 (ArLV1), which we encountered in *A. thaliana* RNA sequencing datasets generated in our laboratories and which was found to be widespread in other datasets obtained from sequence data repositories. We found that plants from several *A. thaliana* accessions, including the widely used accession Col‐0 (CS60000), tested positive for ArLV1. We identified different isolates of the virus across the NCBI Sequence Read Archives (SRAs) and investigated disease symptoms, infectivity, plant growth and effects on the *A. thaliana* transcriptome and on abiotic stress resilience. Regular screening for the presence of this widely present – but unnoted – virus and further investigation of its possible effects is highly relevant for the plant science community working with *A. thaliana*.

## Materials and Methods

### ArLV1 identification and genome assembly

The RNA‐Seq dataset of Kloth *et al*. (2016) was mapped against the TAIR10 *A. thaliana* reference transcriptome (Lamesch *et al*., 2011) with Tophat v.2.0.13 and intron length 20–2000. The dataset from Utrecht University was mapped against the Araport10 reference transcriptome with Kallisto (Bray *et al*., 2016; Methods S1). Unmapped reads were *de novo* assembled in CLC Genomic Workbench v.9 (Qiagen) using standard settings and the resulting contigs were blasted against the NCBI RefSeq database. Contigs showing a clear identity to different comoviruses were retained for further analysis. As per *A. thaliana* genotype (ALL1‐3, Pent‐1, Ep‐0 and Col‐0), we assembled unique contigs, of which the virus isolate in ALL1‐3 from the Wageningen dataset was submitted to GenBank under accession nos. MH899120.1 (RNA1) and MH899121.1 (RNA2), respectively. This Wageningen isolate is later referred to as ArLV1_A, the Col‐0 isolate from Utrecht as ArLV1_B. To identify the phylogenetic position of this virus within the genus *Comovirus*, RefSeq comovirus sequences were downloaded from NCBI and the amino acid sequence of the conserved Pro‐Pol region from RNA1 was aligned using Mafft v.7.475 with the auto option (Katoh & Standley, 2013). Maximum likelihood (ML) phylogeny was reconstructed using IQ‐Tree v.2.0.3 with ModelFinder and 1000 ultrafast bootstrap replicates (Kalyaanamoorthy *et al*., 2017; Minh *et al*., 2020).

### Datamining of NCBI SRAs

Illumina‐generated *A. thaliana* RNA‐Seq datasets (SRAs) were downloaded from the NCBI SRA (https://www.ncbi.nlm.nih.gov/sra) and searched for the presence of ArLV1 RNA1 and RNA2 sequences with the Blastn_vdb program from the NCBI SRA toolkit v.2.9.0 (min. 500 reads per SRA dataset; automatization script available on Zenodo).

### Phylogenetic analysis

From the NCBI SRA output, accessions containing ArLV1 were selected based on the presence of at least 500 reads of RNA2, representing full coverage. From these, one SRA each from 38 randomly chosen accessions was selected, from which the consensus nucleotide sequences of RNA1 and RNA2 were retrieved by reference mapping (CLC Genomic Workbench v.20) against the ArLV1 sequences (MH899120.1 (RNA1) and MH899121.1 (RNA2), respectively) with options ‘low coverage definition threshold = 3’ and ‘insert N‐ambiguity symbol’. The ML phylogenies were reconstructed using the conserved Pro‐Pol region from RNA1 (Le Gall *et al*., 2008) and ORF2 from RNA2 of these 38 consensus sequences together with four sequences from different *A. thaliana* ecotypes from Wageningen and Utrecht (ALL1‐3_Wageningen, Pent‐1_Wageningen, Ep‐0_Wageningen and Col‐0_Utrecht) using IQ‐Tree v.2.0.3 with ModelFinder and 1000 ultrafast bootstrap replicates (Kalyaanamoorthy *et al*., 2017; Minh *et al*., 2020). Evidence of recombination was assessed using the Phi test implemented in SplitsTree (Bruen *et al*., 2006; Huson & Bryant, 2006). A world map of ArLV1 occurrences, based on available GPS data (https://1001genomes.org) for 36 accessions with available latitude and longitude coordinates, was made with the R package ggplot2. Isolation by distance was assessed using the Mantel test implemented in the R package ade4, where distance was calculated as Euclidean distance from the coordinates using the R package sp and genetic distances were estimated using IQ‐Tree.

### ArLV1 inoculations

Arabidopsis latent virus 1 inoculum was obtained by harvesting leaf material from ArLV1‐positive *A. thaliana* plants and grinding it (1 : 1, w/v) in an inoculation buffer (0.03 M phosphate buffer, pH of 7.2). For inoculation, leaves of *A. thaliana* plants in leaf stage 8–10, or *Nicotiana benthamiana* plants in leaf stage 5–8 were lightly dusted with carborundum powder, and inoculum was applied by gentle rubbing. Plants were rinsed with water 10 min after inoculation. Virus symptoms were assessed visually at 7–10 d post‐inoculation (DPI). Presence of virus in inoculated plants was checked by SYBR Green quantitative reverse transcription polymerase chain reaction (RT‐qPCR) and transmission electron microscopy (TEM) with a JEM1400 Plus (Jeol, Nieuw Vennep, the Netherlands) using a leaf dip assay according to standard protocols (Hayat & Miller, 1990).

### Virus detection by quantitative RT‐PCR (RT‐qPCR)

For a detailed step‐by‐step protocol of how samples were collected and used in RT‐qPCR, refer to Fig. S1. In addition to this protocol, at least two primer pairs were used per sample: one for RNA1 (RNA1_1; ArLV‐RNA1‐Fw: TCTGCCAGTACTGGAGAGG and ArLV1‐RNA1‐Rv: GTCATCCAACAAATAGGAAC) and one for RNA2 (RNA2; ArLV1‐RNA2‐Fw: CACCAATAACACCCCAAAA and ArLV1‐RNA2‐Rv: GCATTTCCACAGAGTCTCG). For the seed transmission experiments, an additional primer pair for RNA1 was used (RNA1_2; ArLV1‐RNA1‐Fw: TGTCGTGATAACTGATGG and ArLV1‐RNA1‐Rv: CTAACCTCTTTCCTCCCC). ΔC_t_ values were calculated per primer pair by subtracting the average control C_t_ values from the same RT‐qPCR run. If multiple primer pairs for RNA1 were used, the average delta CT of the two was calculated and used in visualization. K‐means clustering was used to separate the positive samples from the negative samples.

## Results

### Identification of ArLV1

**Fig. 1:**
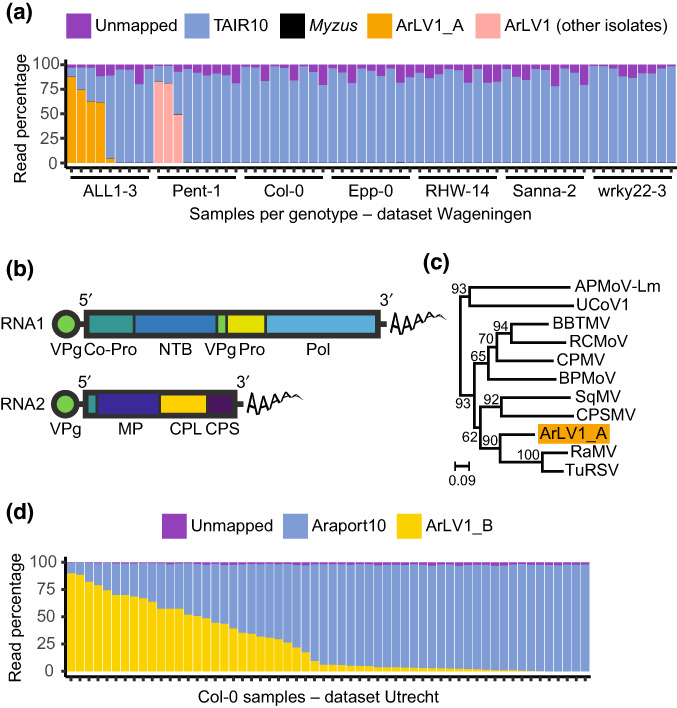
Occurrence and identification of Arabidopsis latent virus 1 (ArLV1) in *Arabidopsis thaliana*. (a) Mapping percentages of the reads in the Wageningen RNA‐Seq dataset. As this dataset involved leaf material from both naïve and aphid‐infested samples (Kloth *et al*., 2016), reads of *Myzus persicae* aphids were included in our analysis as well, but only a few were identified. Sequence information for further analyses was obtained from ALL1‐3, and the virus isolate was named ArLV1_A. (b) Schematic representation of RNA1 and RNA2 of a typical comovirus, adapted from King *et al*. (2012). Co‐Pro, proteinase cofactor; CPL and CPS, large and small capsid proteins; MP, movement protein; NTB, NTP‐binding proteins; Pol, RNA‐dependent RNA polymerase; Pro, proteinase; VPg, genome‐linked viral protein. (c) Maximum likelihood tree of the translated Pro‐Pol region from RNA1 of ArLV1_A (the isolate from ALL1‐3) and 10 other comoviruses: APMoV‐Lm (Andean potato mottle virus; MN176101), UCoV1 (Ullucus virus C; MH645163), BBTMV (broad bean true mosaic virus; NC_022004), RCMoV (red clover mottle virus; NC_003741), CPMV (cowpea mosaic virus; NC_003549), BPMoV (bean pod mottle virus; NC_003496), SqMV (squash mosaic virus; NC_003799), CPSMV (cowpea severe mosaic virus; NC_003545), RaMV (radish mosaic virus; NC_010709) and TuRSV (turnip ringspot virus; NC_013218). The substitution model LG + G4 was selected based on the Bayesian information criterion. Branch lengths (scale) represent amino acid substitutions per site. Note that this is an unrooted tree. (d) Mapping percentages of the ArLV1_B reads in the Utrecht RNA‐Seq dataset from *A. thaliana* accession Col‐0, involving leaf samples from an abiotic stress experiment (combinations of mild drought, high temperature and submergence; see Methods S1 and Morales *et al*., 2022).

An analysis of RNA‐Seq transcriptome datasets from several natural *A. thaliana* accessions, some of which were part of a study by Kloth *et al*. (2016), hereafter referred to as the ‘Wageningen’ dataset, showed for some samples an unexpectedly low mapping percentage of plant reads (as low as 17.4%) in the alignments to the TAIR10 *A. thaliana* reference transcriptome (Lamesch *et al*., 2011; Fig. 1a). *De novo* assembly of the unmapped reads identified two contigs with lengths of 5953 and 3600 nucleotides, displaying a segmented genome organization typical for viruses from the genus *Comovirus*, family Secoviridae, order Picornavirales (Thompson *et al*., 2017; Fig. 1b). To assess the phylogenetic position of this comovirus, we compared the amino acid sequence of the highly conserved Pro‐Pol region of RNA1 with the Pro‐Pol region of other comoviruses (Le Gall *et al*., 2008). The highest degree of nucleotide identity of this comovirus is 68% with *Radish mosaic virus* (RaMV; NC_010709). This analysis clearly identified the virus as a distinct comovirus (Fig. 1c). Samples of two *A. thaliana* accessions contained extremely high numbers of reads from the newly identified comovirus: accession ALL1‐3 (CS76090) up to 88.2% (isolate ArLV1_A), and accession Pent‐1 (CS76209) up to 83.0%. We also identified an extremely high abundance of similar RNA1 and RNA2 reads from the same comovirus in some, but not all, RNA‐sequenced samples of accession Col‐0 at Utrecht University, with a mapping percentage of up to 90.08% of viral reads (isolate ArLV1_B) (Fig. 1d). The occurrence of the virus in samples was not linked to the applied treatments (neither abiotic stress nor aphids). As this comovirus does not seem to cause any apparent visible symptoms in any of the *A. thaliana* accessions in our studies, we named the virus Arabidopsis latent virus 1 (ArLV1).

### ArLV1 detection, inoculation and transmission

**Fig. 2:**
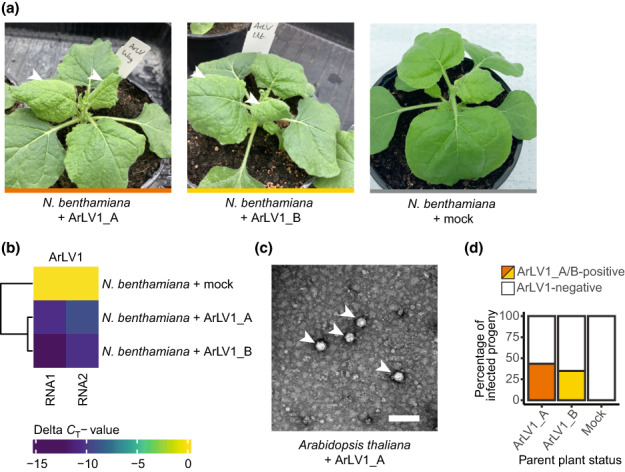
Mechanical inoculation, detection and seed transmission of Arabidopsis latent virus 1 (ArLV1). (a) *Nicotiana benthamiana* plants at 2 wk after mechanical inoculation with two isolates of ArLV1 – ArLV1_A (left photo) and ArLV1_B (middle photo) – both showing ArLV1‐induced leaf mottling symptoms (indicated with arrowheads). The right photo shows a control plant mock inoculated with buffer. (b) Quantitative reverse transcription polymerase chain reaction (RT‐qPCR) detection of RNA1 and RNA2. qPCR detection of ArLV1 in *N. benthamiana* was repeated as a positive control every time we inoculated *Arabidopsis thaliana*, with comparable results. (c) ArLV1_A in infected *A. thaliana* leaves, visualized by transmission electron microscopy. Arrowheads indicate the viral particles (Bar, 100 nm). (d) Percentage of infected progeny from *A. thaliana* Col‐0 parent plants infected with the isolate ArLV1_A or ArLV1_B. Infection was detected by RT‐qPCR on leaf material (Figs S1, S2).

To assess possible plant infections, we developed and validated a RT‐qPCR suitable for plant leaf material (Fig. S1). Arabidopsis latent virus 1 isolates could be mechanically inoculated from infected *A. thaliana* plants, grown from infected seed batches, to *N. benthamiana*, known for its susceptibility to many plant viruses (Goodin *et al*., 2008) and healthy *A. thaliana* plants. *Nicotiana benthamiana* plants showed symptoms of leaf mottling and mosaic patterns at 5–7 DPI (Fig. 2a) and all inoculated plants tested positive when compared with mock‐inoculated plants (Fig. 2b). *Arabidopsis thaliana* plants infected with ArLV1 never showed any visible symptoms and could not be visually distinguished from healthy plants. However, transmission electron microscopy of ArLV1‐infected *A. thaliana* leaf extracts did show typical comovirus particles (Fig. 2c). We used *N. benthamiana* leaf tissue to mechanically inoculate *A. thaliana* Col‐0 with the isolates ArLV1_A and ArLV1_B, with an efficiency of 88% (79/90) (Fig. S2). Taken together, we can state that ArLV1 can be mechanically transferred and has the ability to infect both *N. benthamiana* and *A. thaliana*. As some comoviruses can be efficiently transmitted via seeds (Gergerich & Scott, 1996), we tested the progeny of four different ArLV1‐infected *A. thaliana* Col‐0 parent plants for the presence of ArLV1. In total, 39.1% of the 46 plants grown from these seed batches tested positive for ArLV1 (Figs 2d, S3), indicating seed transmission of ArLV1.

### Occurrence and phylogeny of ArLV1

**Fig. 3:**
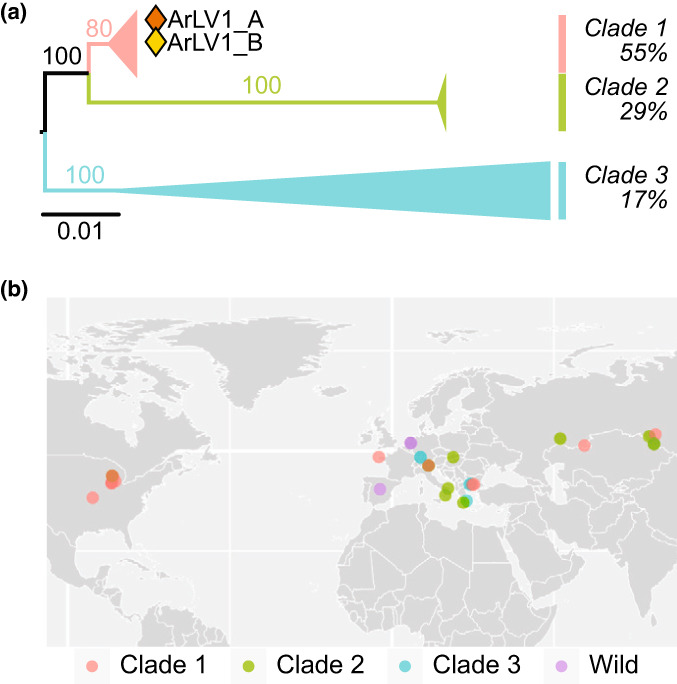
Phylogeny and geographic information of different Arabidopsis latent virus 1 (ArLV1) isolates. (a) Collapsed maximum likelihood tree of the nucleotide sequences of ORF2 on RNA2 of 38 ArLV1 isolates. Branch lengths (scale) represent nucleotide substitutions per site. (b) World map with the coordinates of 36 different ArLV1 isolates, colored according to their RNA2 clade. For the ArLV1 isolates collected outside of the laboratory environment (wild), we do not have clade data available. Genotypes from *Arabidopsis thaliana* accessions collected at relatively close proximity are represented by overlapping dots (brown dots represent sequences from both clade 1 and clade 2).

To reveal if ArLV1 is also present in *A. thaliana* datasets other than the Wageningen and Utrecht datasets, we initiated a search in publicly available *A. thaliana* RNA‐Seq datasets. Out of a total of 6477 RNA‐Seq datasets analyzed, 547 (8.45%) contained at least 500 reads mapping to RNA1 and 500 reads mapping to RNA2 of ArLV1, indicating a full coverage of the viral genome. Altogether, ArLV1 was detected in 176 out of 711 accessions (24.75%) in the public datasets analyzed. Arabidopsis latent virus 1 RNAs could not be detected through reference mapping in a randomly selected set of 35 SRAs from the related species *Arabidopsis lyrata*. Therefore, the precise host range of ArLV1 remains to be determined. To assess the genetic diversity of ArLV1, nucleotide sequences from the Pro‐Pol regions of RNA1 and from the full open reading frame of RNA2 of four isolates from different *A. thaliana* ecotypes from Wageningen and Utrecht and 38 different NCBI‐derived datasets, each with a full coverage of RNA1 and RNA2, were used for phylogenetic analyses (Figs 3a, S4, S5). The resolved phylogenetic trees supported a separation of isolates in three distinct clades. Clade 1 was represented by 55% of the isolates and occurred across different continents, and clades 2 and 3 were represented by 29% and 17% of the isolates, respectively, and originated from Eurasia, except for one isolate of clade 2 from the US (Pent‐23) (Fig. 3b; Table S1). The isolates within clades 1 and 2 were highly similar, whereas six isolates in the third clade were more divergent (Figs S4, S5). Notably, the phylogenies showed signs of reassortment (e.g. accessions Fell2‐4 and Pent‐23 fall in clade 1 for RNA1, and in clade 3 and clade 2, respectively, for RNA2) (Figs S4, S5). We also detected recombination within each alignment (Phi test *P* < 0.01 for each alignment). RNA1 showed evidence of isolation by distance (*P* = 0.0083, Mantel test), but not RNA2 (*P* = 0.15, Mantel test), leaving it inconclusive as to whether limited geographical dispersal played a role in diversification. To find out if ArLV1 was also present in wild *A. thaliana* populations, we sampled 27 different plants in Arnhem, Wageningen and Woerden (the Netherlands), where the presence of ArLV1 outside of the laboratory environment was confirmed in independent RT‐PCR assays for eight out of 27 plants (Fig. S6). In addition, the presence of ArLV1 in wild *A. thaliana* plants from Ciruelos de Coca and Carbonero (Spain) (Pagán *et al*., 2010) was confirmed by RT‐PCR and Sanger sequencing confirmation (C. Carrasco‐López & F. García‐Arenal, pers. comm.).

### Analysis of ArLV1 effects

**Fig. 4:**
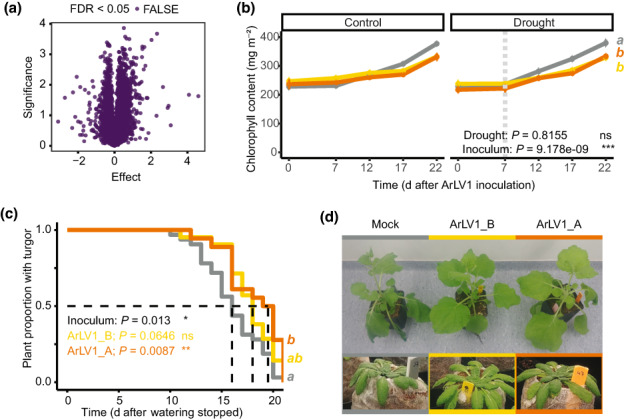
Transcriptomic and phenotypic effects of Arabidopsis latent virus 1 (ArLV1). (a) *Arabidopsis thaliana* Col‐0 transcriptome analysis of four samples with high ArLV1_B (Utrecht isolate) read mapping (average = 81.6%) compared with three samples with low ArLV1_B read mapping (average = 4.18%). (b) Chlorophyll content of the eighth true leaf, following ArLV1 infection in plants subjected to drought (right panel) or kept in well‐watered control conditions (left panel). Inoculation with ArLV1_A (orange), ArLV1_B (yellow) or mock virus (gray) occurred at 0 d post‐inoculation (DPI) and drought was applied at 7 DPI (vertical dotted bar) (see Methods S1). The experiment was repeated twice with similar results (*n* > 20). Both repeats were included in the statistical analysis using mixed linear models with repeats as a random variable. The *P*‐values of ‘inoculum’ or ‘drought’ represent the effect of inoculation with either ArLV1_A, ArLV1_B or buffer (mock), or the effect of the application of drought. (c) Kaplan–Meier survival curve of the fraction of plants that maintained turgor after watering was stopped (at 0 d). The log‐rank *P*‐value for the effect of inoculum is given as well as the Cox regression values for the two ArLV1‐inoculated groups vs mock virus. The experiment was repeated twice and results were combined for visualization and analysis. For experiments (b, c) an alpha of 0.05 was used (different letters (a, b) represent significant differences). (d) Image showing representative *Nicotiana benthamiana* and *A. thaliana* plants inoculated with ArLV1_A, ArLV1_B or mock virus displaying wilting after 4 d (*N. benthamiana*) or 15 d (*A. thaliana*) of water deprivation.

Although ArLV1 produces no visible symptoms in *A. thaliana*, we wanted to study possible effects that ArLV1 infection may have on the transcriptome responses of *A. thaliana* plants. To this end, we compared the transcriptomes of seven samples from the Utrecht transcriptome analysis (Methods S2). This dataset included four samples with high numbers of viral reads (a mapping percentage of 78.94–90.08%) and three samples of plants of identical age and growth conditions, but with low numbers of viral reads (a mapping percentage of 0.01–9.56%). We did not find any significant differentially expressed *A. thaliana* genes between the two groups (Fig. 4a). However, we did observe a slight but significantly lower Chl content in plants inoculated with ArLV1 compared with mock‐inoculated plants, regardless of the availability of water (Fig. 4b; Methods S3). Other morphometric traits, such as leaf number or leaf surface area, were unaffected by the virus (Fig. S7). When watering was stopped to induce drought conditions, mock‐inoculated plants wilted significantly a few days earlier than ArlV1‐inoculated plants (Fig. 4c). The same phenotype was observed for *N. benthamiana* plants subjected to similar drought conditions (Fig. 4d). Next, we quantified the area of the pectin mucilage layer present on the external surface of seeds. The presence of this layer is associated with seed longevity and the size and integrity are known to be altered by several viruses (Bueso *et al*., 2017; Methods S3). Although the different growing conditions in Wageningen and Utrecht significantly (*P* = 0.004) affected the area of the pectin mucilage layer, ArLV1 infection of the parent plant did not affect the size of the mucilage layer (*P* = 0.147) (Fig. S8a). Likewise, *A. thaliana* Col‐0 plants inoculated with the two isolates of ArLV1 or mock‐inoculated did not differ from each other in the onset of flowering (Fig. S8b), suggesting that ArLV1 is not affecting these fitness parameters. Taken together, these results indicate that while ArLV1 is mainly a latent virus to *A. thaliana*, some minor phenotypic effects can be observed, such as lower Chl content and improved drought tolerance. The latter effect was observed in both *A. thaliana* and *N. benthamiana*.

## Discussion

The advent of high‐throughput sequencing technologies has greatly contributed to the understanding of the concept of holobionts (Nobori *et al*., 2018), which includes not only the study organism itself but also its associated communities (Hassani *et al*., 2018). In contrast to the use of microarrays, high‐throughput sequencing can reveal the presence of unexpected and unrevealed organisms and biological agents in study systems (Vandenkoornhuyse *et al*., 2015), especially for viruses (Massart *et al*., 2014; Maclot *et al*., 2020). As sequencing has become more affordable and prevalent, opportunities arise to uncover the unknown metagenomes of our study systems. This may reveal critical microbial factors that potentially influence plant (physiological) processes, and therefore encourages us to investigate unexpected results, such as those illustrated here.

In this study, Illumina‐derived RNA‐Seq datasets from *A. thaliana* transcriptome studies with exceptionally low numbers of plant‐specific reads revealed the presence of an as yet uncharacterized plant virus belonging to the genus *Comovirus*. Given its apparent latent nature in *A. thaliana*, we named this virus Arabidopsis latent virus 1 (ArLV1). We showed the infectivity of this newly discovered virus for *A. thaliana* and *N. benthamiana* and its high transmissibility to *A. thaliana* progeny via seeds from ArLV1‐infected plants. The TEM studies confirmed typical comovirus particles in infected *A. thaliana* plants and a RT‐qPCR test was developed that allows detection of the virus in plant material.

From the nearly 6500 public *A. thaliana* SRAs that were tested, 8.45% contained evidence of an ArLV1 infection, accounting for near 25% of the ‘natural’ *A. thaliana* accessions and some mutant lines in the dataset. This indicates that ArLV1 is present in the *A. thaliana* stocks of laboratories worldwide. We also confirmed its presence in wild *A. thaliana* populations, both in the Netherlands and in Spain. A total of 38 *A. thaliana* accessions were selected for a phylogenetic analysis of ArLV1 sequences, including the widely used accession Col‐0. The phylogenetic analysis divided these virus sequences into three clades with different geographical distributions, where occasional recombination and re‐assortment also occur. This, in addition to its apparent latent nature, its occurrence in plants directly collected from the wild and a large number of laboratory stocks of *A. thaliana* accessions collected from many geographical regions, clearly suggests that ArLV1 is a virus that has been naturally associated with *A. thaliana* for a long period of time.

Our RNA‐Seq datasets obtained from different *A. thaliana* accessions in independent studies in both Wageningen and Utrecht show that an ArLV1 infection in *A. thaliana* can result in > 90% of virus‐specific reads. This is an indication that ArLV1 can potentially reach very high titers in infected plants. Interestingly, these large differences in ArLV1‐specific read numbers (both absolute and relative to the total read count) were observed in datasets from both Wageningen and Utrecht, between individual plants from the same accession grown and processed in the same experiment. In addition, RT‐qPCR results for individual plants grown under the same conditions varied between ΔC_t_ values of −20 and −6. The two laboratories in Wageningen and Utrecht used different plant growth conditions (Methods S1), making it unlikely that specific growing conditions or sampling and/or sample processing biases are related to these differences. The reason(s) for the high variation in ArLV1 virus titers remain to be elucidated. Although we did not identify public datasets with high amounts of virus‐specific reads, we suspect that unpublished *A. thaliana* transcriptome studies possibly also contain comparable high numbers of ArLV1 reads, but have not been reported and made available owing to the low number of plant‐specific reads.

Although a direct comparison of the transcriptomes of *A. thaliana* plants with high and low viral infections did not reveal differentially expressed genes, ArLV1 does seem to have a small but significant positive effect on drought resilience, putatively via virus‐induced reduced stomatal conductance (Pasin *et al*., 2020; Manacorda *et al*., 2021). In addition to the obvious negative impacts on plant morphology, physiology and yield (Prasad *et al*., 2020), viruses are also known to affect their host positively in their tolerance to abiotic stress (Gorovits *et al*., 2019; Rahman *et al*., 2021; Aguilar & Lozano‐Duran, 2022). Some viruses even change from parasitic to mutualistic with a change in environmental conditions (González *et al*., 2021). The protective effect of plant viruses is mainly observed for drought (Mishra *et al*., 2021), but some studies also report that viruses can have a positive effect on plant responses to (other) abiotic stresses, such as high temperature (Anfoka *et al*., 2016) or salt stress (Sinha *et al*., 2021). To our knowledge this is the first example of a comovirus exerting such a positive effect. Further research is needed to reveal the underlying molecular mechanisms of how ArLV1 can affect plants’ tolerance to drought, and to determine if similar effects can be observed for other abiotic stressors.

Taken together, we have identified an as yet unknown comovirus that is widely distributed in *A. thaliana* in laboratories and in the wild. The high prevalence in transcriptome datasets and its high potential for seed transmission make it safe to assume that ArLV1 will be present in research setups with the model species *A. thaliana*, with a significant part of the plants within a given experiment unknowingly infected with ArLV1. This may have unknown consequences for the interpretation of data obtained from these studies. Given its mainly latent nature, ArLV1 has probably remained unnoticed for a long time, and through its efficient seed transmission has spread worldwide. Its prevalence and lack of obvious disease symptoms make ArLV1 a plant virus that needs to be treated with scrutiny. We recommend routine screening to detect the presence of ArLV1 in seed stocks of *A. thaliana* and possibly related species in laboratories and public repositories. The virus can be easily detected in plant samples via the RT‐qPCR‐based method described in Fig. S1. This will permit rapid selection of ArLV1‐free plants and seed batches before proceeding with experiments, thus preventing potential confounding effects of the virus.

## Competing interests

The authors declare there are no competing interests.

## Author contributions

AV, KJK and RAAV designed the experiments; AV, KJK, RAAV, AK, GHO, KR, ZJ, JD and CD performed the experiments; AV, KJK and RAAV analyzed the results; and AV, KJK, RAAV, RS and MZ wrote the paper.

## Supporting information


**Fig. S1** Protocol for ArLV1 detection in plant material.
**Fig. S2** *Arabidopsis thaliana* inoculation efficiency.
**Fig. S3** *Arabidopsis thaliana* seed transmission of ArLV1.
**Fig. S4** Phylogenetic tree of the Pro‐Pol region from ArLV1 RNA1.
**Fig. S5** Phylogenetic tree of the full ORF from ArLV1 RNA2.
**Fig. S6** ArLV1 RNA2 PCR fragments of wild collected *Arabidopsis thaliana*.
**Fig. S7** Leaf number and leaf surface area of plants subjected to ArLV1 inoculation.
**Fig. S8** Seed mucilage layer and flowering time are not affected by ArLV1.
**Methods S1** RNA‐Seq approaches and plant growth, treatment and sampling conditions.
**Methods S2** Transcriptomic analysis of *Arabidopsis thaliana* Col‐0 accession.
**Methods S3** Phenotypic parameters.
**Table S1** *Arabidopsis thaliana* Sequence Read Archive datasets included in the geographical analysis.
Please note: Wiley Blackwell are not responsible for the content or functionality of any Supporting Information supplied by the authors. Any queries (other than missing material) should be directed to the *New Phytologist* Central Office.

## Data Availability

The sequences of the ArLV1_A isolate in ALL1‐3 from the Wageningen dataset are available at NCBI (www.ncbi.nlm.nih.gov/) under accession nos. MH899120.1 (RNA1) and MH899121.1 (RNA2), respectively. The Python scripts used for ArLV1 detection in the NCBI‐SRA have been deposited in Zenodo (https://zenodo.org/record/6834314#.YtA3ei8Rqys). RNA‐Seq datasets were deposited at NCBI under project nos. PRJNA858638 (Wageningen dataset) and PRJNA863409 (Utrecht dataset). All other data are available from the corresponding author upon request.
